# Lord Lister: X. His Search for Three Ideals

**Published:** 1918-03-30

**Authors:** 


### LORD LISTER.

### X.—His Search for Three Ideals.

To test the validity of his reasoning, Lister tied the carotid artery of an old horse that had passed the usual limit of equine longevity, conducting the operation, of course, with all the precautions that we now call aseptic, but that he called antiseptic, and using for ligature a piece of purse-silk that had been steeped in a saturated watery solution of carbolic acid. The ends of the ligature were not left long and hanging out of the wound, as the previous practice had always been, but were cut short, and the wound was closed over them. The wound healed by first intention, and, when the horse died of old age some six weeks afterwards, Lister found, on dissection, that the silk was unchanged, but was encapsuled in fibrous tissue.

The result was not all that had been hoped. The ligature had not been eaten away by the granulations, but at least it had lain, like a bullet or a fragment of glass, innocuous, and not a provocative of suppuration. Though the result was not perfect, it was a most momentous improvement on anything that had been effected before, and it encouraged Lister to proceed in the same way in an operation on the human body. An opportunity soon occurred, and he tied the external iliac artery in an elderly lady, with the same precautions that he had used in tying the carotid of the horse. The operation was completely successful. The lady made a perfect recovery from it, and lived for ten months after it. On her death from another aneurism, the site of the operation was examined, and the result was found partly to justify Lister's anticipations, partly to disappoint them. The loop of the silk ligature had disappeared, consumed by the granulations; but the knot and the two ends, though now shorter than they had been left at the operation, remained, and this portion was found encapsuled in a tiny cavity containing a minute quantity of what looked like yellow pus, though on examination it was found not to be pus.

As he felt certain that the silk had been rendered thoroughly antiseptic, or as we should now say sterile, the non-absorption of this part of the ligature was something of a disappointment to Lister, and he attributed it, no doubt correctly, to the material of which the ligature was composed, and set himself to find some other material that should be less resistant to the eroding and consuming power of the granulations. The search was long, and involved innumerable experiments and trials. As the substance must be of an organic nature, and preferably of animal rather than vegetable origin, the first substance that suggested itself was catgut, and catgut was tried with success. Catgut, however, like all things in this sublunary world, is imperfect. It has its disadvantages. It is stiff, and difficult to tie in a tight knot. It is elastic and slippery, and apt to become loose or altogether untied when it has been tied. It is hard, and difficult to penetrate by antiseptics. Different specimens of it differ much in the rapidity with which they undergo absorption. Some, unhappily, swelled up, became gelatinous, and lost their constricting power before the artery had had time to become permanently obliterated. All these defects Lister set himself to remove, and his efforts were continued in a long course of experimentation that lasted to the very end of his active life. This alone is evidence that he was never able to prepare a ligature that satisfied his ideal of perfection, or rather, it would be more accurate to say he was never confident of being able to produce such a ligature; for, owing to subtle differences in the composition of catgut, the very same treatment that produced from one specimen a perfect ligature produced from another a ligature that was imperfect in some important particular—that was too persistent, or too evanescent, or too resilient, or too brittle, or too difficult to sterilise, or that had some other defect.

The introduction of the catgut ligature was, however, in spite of all its defects, the crowning achievement of Lister's life. It enabled him to abolish at once and for ever the practice of leaving the ends of the ligatures hanging out of the wound. It enabled him, therefore, to close the wound completely on the operation table, and to expect complete healing by first intention in a few days or even hours, instead of watching with anxiety for days, sometimes for more than a week, lest infection should creep up the course of the ligatures and set up in the wound suppuration or something worse. Next to the observance of surgical cleanliness, in which expression both asepsis and antisepsis were included, the greatest improvement in surgery since the introduction of the ligature was the substitution of the catgut ligature with its ends cut short for the yarn ligature with its ends, or one end, left long and hanging out of the wound. The catgut ligature did this, and much more than this. It rendered the operation of ligation of arteries in their continuity, instead of a frightfully hazardous operation, the result of which it was impossible to foresee, one of the simplest and safest operations of surgery.

Carbolic acid, in spite of its defects, maintained its position as the most potent and trustworthy antiseptic for many years; but Lister was never satisfied with it. He was never satisfied with anything that fell short of perfection. He was constantly seeking for some germicide that should be as effectual as carbolic acid, but should be free from its defects. Perfection is rare in this world, and Lister never found all that he sought for, nor has it been discovered since his day. The search is still proceeding, and during the war that is now in progress many new antiseptics have been tried, and have gone out of use or remained in use according to the results of the trials. So much ignorance

☞ Previous articles appeared Jan. 26, Feb. 2, 9, 16, 23, March 2, 9, 16, and 23, p. 525.

**LORD LISTER—(*continued*).**

of the history of Listerism and of its principles still prevails, that it has been thought and asserted that the use of these novel antiseptics is a departure from Listerism, and their adoption has even been spoken of as an improvement greater than Listerism, and one that is to supersede Listerism. Such assertions display the profound and humiliating ignorance of those who make them. He who succeeds in finding a perfect antiseptic, which shall be deadly to pathogenic microbes and harmless to the living tissues of the patient, will have effected the greatest improvement in Listerism that has been effected since Lister's day; but he will no more have improved upon Lister's principles or departed from them than the man who devises glass of a new composition, or a more accurate method of grinding lenses, improves on the principles of the telescope, or departs from the principles of Galileo and Newton. Only the grossly ignorant or the intentional falsifier says, or ever did say, that Listerism is bound up with the use of carbolic acid.

Side by side with his researches and experiments for the discovery of the ideal antiseptic and the ideal ligature, Lister was continually searching for the ideal dressing, the medium that should maintain an effectual barrier against the contamination of wounds by holding against them an effectual germicide, surrounding them with an aseptic atmosphere, and sterilising the discharges, if any, as they escaped. The earliest antiseptic dressing was a piece of lint, dipped in a strong solution of carbolic acid, and laid upon the wound. The carbolic acid injured the skin, produced inflammation in it, and soon produced a raw surface eminently favourable to the culture and multiplication of pathogenic microbes. The substitution of carbolised oil for carbolised water was a great improvement. The oil was more bland, less irritating, and less serious in its effects, but it was far from perfect. It had in minor degree the defects of the watery solution. Then Lister devised a putty, made of whitening combined with watery or oily solution of carbolic acid, and spread, first upon block-tin, which is plastic, and can be moulded to the surface it is to cover, while it is at the same time impervious and prevents the dressing from becoming dry and the carbolic acid from evaporating. The tin was soon superseded by a double layer of muslin, between the layers of which the putty was spread as a poultice. Whatever the dressing, it was made of such a size as to extend in every direction considerably beyond the margins of the wound, so that any escaping discharge would be in contact for some length of its course with the carbolic acid, would become impregnated with it, and so incapable of forming a nidus for the development of microbes.

These impermeable dressings were faulty, in that the skin beneath them became sodden and sometimes sore. An improvement was effected by substituting oakum—a mass of loose fibre impregnated with Stockholm tar. This received and absorbed the discharges and at the same time rendered them aseptic; but it was sticky, messy, and odorous. Lister set himself to discover, first, a convenient vehicle for the antiseptic; second, a suitable fabric to hold it; and, third, a method of distributing the vehicle containing the antiseptic evenly among the fibres of the fabric. The search was long, and involved a long course of experimentation for which it seems incredible that a busy surgeon to a large hospital, with a large private practice, and a professor of surgery to boot, could have carried on simultaneously with all his other work; but Lister had the enthusiasm that left him no rest, and the belief in the attainment of his ideal that inspired him, and eventually he chose resin diluted with paraffin as his vehicle and gauze as his fabric. The latter holds the field even to this day. Carbolic acid remained for many years the sole antiseptic, and, having attained the astonishing measure of success with it that he did attain, any man but Lister would have been content with it and would have sought no farther. But Lister was never content with anything short of perfection. He was continually experimenting and improving his methods and his materials, and up to the end of his long active life he was still seeking the ideal antiseptic. The search is still proceeding.

(*To be continued.*)

